# Superior mediastinal mature cystic teratoma with gastrointestinal adenocarcinoma transformation: Report of a case

**DOI:** 10.18632/oncotarget.9532

**Published:** 2016-05-21

**Authors:** Chen Lin, Yiqun Du, Yuan Li, Huijie Wang, Jianhua Chang

**Affiliations:** ^1^ Department of Medical Oncology, Fudan University Shanghai Cancer Center, Shanghai, 200032, P.R. China; ^2^ Department of Pathology, Fudan University Shanghai Cancer Center, Shanghai, 200032, P.R. China

**Keywords:** germ cell tumors, teratoma with malignant transformation, FOLFIRI chemotherapy regimen, whole exome sequencing

## Abstract

Presented herein is a case of mediastinal mature teratoma with adenocarcinomatous transformation predominantly composed of mucinous adenocarcinoma in a 25-year-old man. Disease progressed despite application of surgical removal, adjuvant radio- and chemotherapy. Further immunohistochemical stains indicated a gastrointestinal origin of the tumor. Consequently chemotherapy according to the FOLFIRI regimen was applied that resulted in good response. To our knowledge, this is the first report of a clinical remission from chemotherapy with the FOLFIRI regimen after comprehensive initial treatment with surgery, radio- and chemotherapy for a patient with teratoma with malignant transformation, highlighting the importance of choosing an appropriate chemotherapy regimen.

## INTRODUCTION

Germ cell tumors (GCTs) account for approximately 15% of mediastinal tumors in adults [1]. Mediastinal GCTs comprise diseases ranging from benign encapsulated tumors to extremely aggressive and invasive neoplasms, with teratoma being the most common type. Teratomas are defined histologically as containing tissues derived from all three germ layers, namely ectoderm, mesoderm and endoderm. Mediastinal mature teratoma is a benign tumor that shows well-differentiated somatic elements, such as skin, fat, nerve, and cartilage. Malignant teratoma comprises three histological forms: immature teratoma, teratoma with other malignant germ cell tumor components, and teratoma with malignant transformation (TMT) [2]. Occasionally, teratomas may undergo malignant transformation in which non-germ cell malignancies emerge. Little is known about the incidence and clinicopathological features of somatic-type malignancies (non-germ cell malignancies) in mediastinal mature teratoma because of its rarity. The prognosis of patients with TMT is poor compared to GCT; however, its management may be improved by adapted chemotherapy [3]. Here, we report a case of a patient with teratoma with malignant transformation in the mediastinum who achieved good response to chemotherapy specific to the transformed histology, rather than teratoma itself.

## CASE REPORT

### Anamnesis

**Figure 1:**
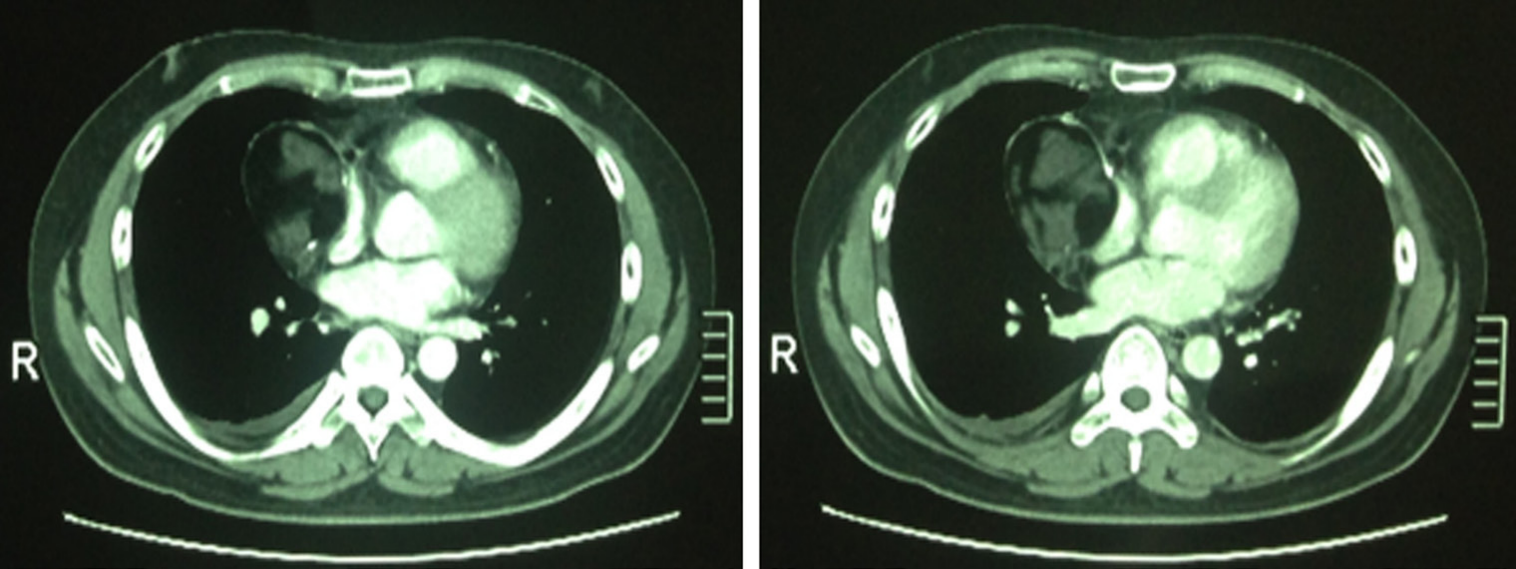
The original chest computed tomography of the patient The CT showed a well-circumscribed cystic and solid mass lesion in the right portion of the superior mediastinum measuring 6 × 5 cm with right pleural effusion.

A previously healthy 25-year-old Chinese man presented to local hospital with complaint of gradually progressive heaviness of chest for three months. There were no associated signs of superior vena cava syndrome, no lymphadenopathy, and no abnormalities on genital examination. His routine hemogram, urine and blood biochemical analyses were within normal ranges. Contrast-enhanced computed tomography (CT) scan (Figure 1) of the chest showed a well-circumscribed 6 × 5 centimeters (cm), cystic and solid mass lesion in the right portion of the superior mediastinum with right pleural effusion. The capsule wall of the tumor was almost uniformly thick. The right lung parenchyma and vena cava were both compressed. No radiologic signs of invasion into the surrounding structures were present. There was no evidence of an eventual primary tumor elsewhere. Among serologic tumor markers, human epididymis protein 4 (HE4), squamous cell carcinoma associated antigen (SCC), carbohydrate antigen 19–9 (CA 19–9), cytokeratin 19 fragment (CYFRA 21–1), and neuron specific enolase (NSE) levels were within acceptable limits, but carbohydrate antigen 72–4 (CA 72–4) level was 36.56 U/ml (normal range [NR]: 0–6.9 U/ml) and carcinoembryonic antigen (CEA) level was 20.42 ng/ml (NR: 0–5.2 ng/ml). Otherwise his medical history was unremarkable.

### Pathologic findings

**Figure 2:**
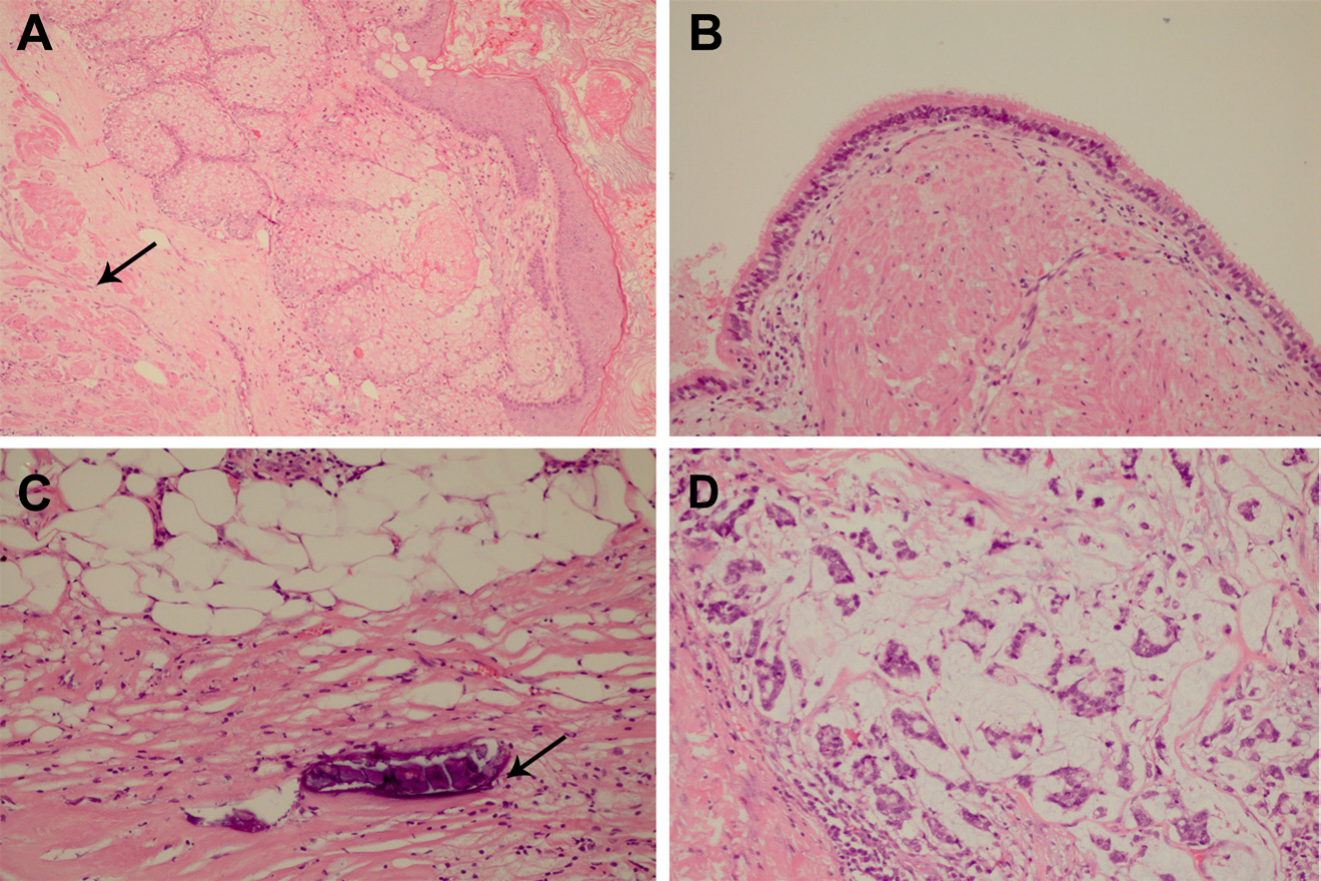
Microscopically, the tumor contained a mature cystic teratoma component with malignant transformation of mucinous adenocarcinoma (H and E, ×200) (**A**) The cyst wall showed keratinizing squamous epithelium, sebaceous glands, and areas of smooth muscle (arrow). (**B**) Pseudostratified ciliated columnar epithelium was observed lining along the internal surface of partial cyst wall. (**C**) In addition, some solid areas exhibited adipose tissue, fibrous tissue, and small foci of calcifications (arrow). (**D**) The malignant transformation of mucinous adenocarcinoma was also identified interspersed between the benign teratomatous elements.

**Figure 3:**
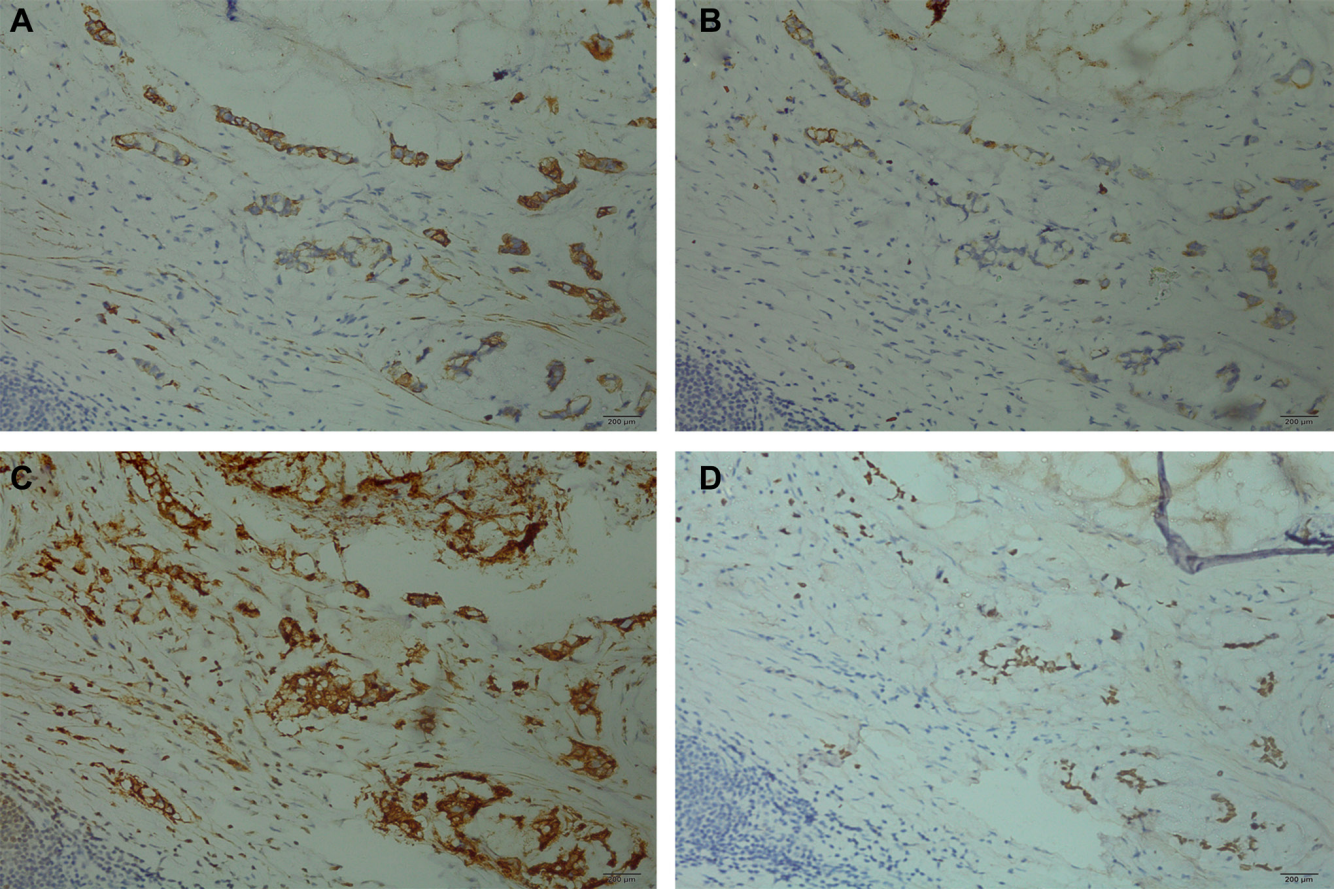
Immunohistochemical staining presented side-by-side on the same tissue (×200) Malignant cells (adenocarcinoma) were positive for CK 7 (**A**), CK 20 (**B**), CEA (**C**), and CDX2 (**D**).

On September 24, 2014, a median sternotomy was performed for complete resection of the mediastinal mass, including a portion of inferior lobe of right lung and thymus. Macroscopically, the mediastinal tumor measured 8 × 6 × 5 cm. On section, it revealed a large multilocular cystic tumor containing brown fluid and sebaceous debris. The cyst lining was thickened with a yellow, gritty surface. From part of the cyst wall, a few grayish-yellow nodular areas were noted, the largest of which measured 2 × 1.5 cm. The cyst did not contain hairs or osteocartilaginous structures and was relatively well demarcated. Microscopically, the tumor contained a mature cystic teratoma component with extensive necroses and regressive calcifications. The cyst wall consisted of keratinizing squamous epithelium and sebaceous glands of ectodermal origin (Figure 2A), and endodermal pseudostratified ciliated columnar epithelium (Figure 2B). In addition, solid areas showed mesodermal derivatives, such as adipose tissue, fibrous tissue (Figure 2C), and smooth muscle (Figure 2A). There were no immature teratomatous elements or other germ cell components. Malignant transformation of adenocarcinoma, chiefly mucinous adenocarcinoma, was also identified interspersed between the benign teratomatous elements (Figure 2D). Moreover, the inferior lobe of the right lung was invaded by mucinous adenocarcinoma, and one right hilar lymph node was positive for metastasis. On immunohistochemistry (IHC), malignant cells (adenocarcinoma) within the tumor were positive for cytokeratin 7 (CK 7), cytokeratin 20 (CK 20), CEA, and caudal-related homeobox gene 2 (CDX 2) (Figure 3), but negative for thyroid transcription factor-1 (TTF-1), calretinin, cytokeratin 5/6 (CK 5/6), Wilms' tumor gene (WT-1) and vimentin (data not shown), supporting the diagnosis of gastrointestinal adenocarcinoma. Consequently, the final pathological diagnosis was mature cystic teratoma with gastrointestinal adenocarcinoma transformation.

### Postoperative treatment

**Figure 4:**
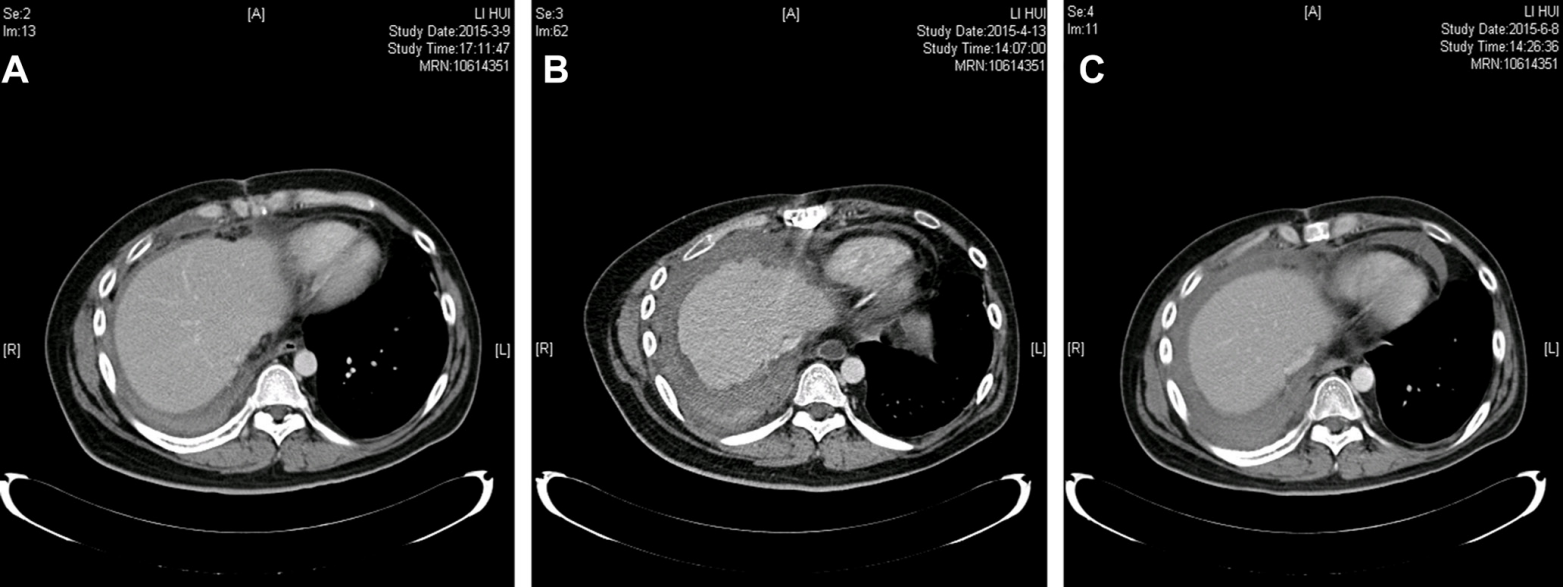
Contrast-enhanced computed tomography (CT) scan of the abdomen (**A**) Peritoneal effusion with multiple peritoneal metastasis. (**B**) Peritoneal metastasis became progressive after one cycle of BEP regimen. (**C**) Radiologically stable disease was achieved after FORFIRI regimen.

Postoperatively, the patient recovered well, his hospital course was unremarkable, and he received two cycles of adjuvant chemotherapy (docetaxel 60 mg/m^2^) and radiotherapy (56 Gy; 5600 cGy/28f confined to the area where the tumor has been removed). Even so, he exhibited peritoneal effusion with multiple peritoneal and mesenteric metastasis nodules on contrast-enhanced abdomen CT scan (Figure 4A) just two months after radiotherapy in March, 2015. Abdominal paracentesis was performed and the cytologic diagnosis from aspirated peritoneal fluid revealed metastatic adenocarcinoma. Then, the patient received salvage chemotherapy according to standard BEP regimen (bleomycin, etoposide, and cisplatin) in a local hospital, but the disease became progressive after one cycle of BEP regimen according to abdomen CT scan (Figure 4B). At the same time, the serum CA 19–9, CA 72–4, CA 242, CA 50 and CEA levels surged to 547.40 U/mL (NR: 0–27.00 U/mL), 300.00 U/mL (NR: 0–6.90 U/mL), 150.00 U/mL (NR: 0–20.00 U/mL), 81.21 IU/mL (NR: 0–25.00 IU/mL) and 41.59 ng/mL (NR: 0–5.20 ng/mL), respectively. Combining the immunohistochemical results, we concluded that the adenocarcinoma originates from gastrointestinal cancer. Therefore, a chemotherapeutic protocol was chosen in line with transformed adenocarcinoma histology and FOLFIRI regimen (irinotecan 180 mg/m^2^ on d1, leucovorin 400 mg/m^2^ on d1 and 5-fluorouracil 400 mg/m^2^ iv bolus d1 followed by 2.4 g/m^2^ as a 46-h continuous infusion starting d1, repeated every 2 weeks) was applied. After a total of 8 cycles of that regimen, the patient achieved clinical response and radiologically stable disease (Figure 4C). The ascitic fluid declined significantly, and a sustained decrease was observed in serologic tumor markers including CA 19-9, CA 242, CA 50 and CEA. The latest test results were 30.70 U/mL, 40.81 U/mL, 11.64 IU/mL and 12.47 ng/mL respectively. At the time of the report, the patient's progression-free survival (PFS) was over 6 months after conclusion of FOLFIRI application. The regimen was well tolerated just causing grade 1 leukopenia as well as mild nausea and fatigue.

## DISSCUSION

TMT denotes a GCT containing a malignant somatic element, like sarcoma, carcinoma or both, which occurs infrequently in around 7% of all mediastinal teratomas [4, 5]. TMT has been divided into two clinical and pathologic conditions: TMT induced by chemotherapy or irradiation and naturally occurring TMT [6]. The former type accounts for nearly all cases of TMT, while the latter type has been reported only rarely.

Surgical resection plays an essential role in therapy particularly for patients with localized disease. No standard adjuvant therapy is recommended since TMT is considered resistant to chemotherapy and radiotherapy [3]. The prognosis of mediastinal GCTs with TMT is extremely poor, especially when metastasis occurs, with a median survival of approximately 9 months [7]. In that advanced setting, adapted chemotherapy based on transformed histology has recently been advocated, which may help improve patients' outcome [5].

To date, four cases of colonic-type adenocarcinoma arising in a teratoma have been reported [4, 7–9]. Two were primary retroperitoneal teratoma while the other two were mediastinal teratoma, all with prominent adenocarcinomatous transformation. However, to our knowledge, our patient is the first one who achieved a response from FOLFIRI chemotherapy regimen after comprehensive initial treatment with surgery, chemo- and radiotherapy. This affirms the importance of selecting a suitable chemotherapy regimen according to the transformed histology because no response to chemotherapy used for treatment of GCTs can be expected. In addition, confirming the origin of adenocarcinoma is vital in this case. Immunostains for gastrointestinal tract markers were essential and other markers like TTF-1 were also applied to exclude adenocarcinoma from respiratory system. On the basis of the IHC profile combined with morphology, the clinical diagnosis of colonic-type adenocarcinoma within the teratoma also depends on the medical history, diagnostic abdominocentesis, image examination, and serum tumor markers.

It is noteworthy that the patient also accepted the whole exome sequencing in Baoteng Laboratory, Shanghai Medicine Center. The correlation analysis of pathogenesis and gene mutation of our patient showed that the most credible mutation sites (top ten) matched with the specific mutation of stomach cancer, colon cancer, lung adenocarcinoma, esophageal cancer and leiomyoma (Table 1), which had a certain reference value to identify the gastrointestinal adenocarcinoma transformation. The gene test also screened out a series of variations associated with pharmacodynamics. For instance, the patient was inclined to be sensitive to gastrointestinal targeted drugs like cetuximab and lapatinib according to the gene analysis (Table 2), indicating a potential to use targeted therapy in case of a relapses after FOLFIRI chemotherapy. Hence, finding out discriminating genes of these kinds of early/late relapses of GCT correlated with the histological types may offer patients opportunities to use targeted drugs.

**Table 1:** Tumor-associated somatic mutations of this patient were screened out after compared with the database of tumor somatic mutation

Gene Name	Location	Standard Gene	Patient's Gene	Histology Message
UNC5A	176295869	CC	CT	Histological type: adenocarcinoma. The primary site:large intestine
GLS2	56867278	CC	CT	Histological type: adenocarcinoma. The primary site:stomach
RPL26	8280962	CC	CT	Histological type: adenocarcinoma. The primary site:cecum
ZNF560	9578436	CC	CT	Histological type:adenocarcinoma. The primary site:colon
S1PR1	101704932	CC	CA	Histological type:squamous cell carcinoma. The primary site:lung
PYHIN1	158914690	GG	GA	Histological type:adenocarcinoma. The primary site:lung
MMP27	102563701	CC	CT	Histological type:adenocarcinoma. The primary site:lung
ZNF99	22940877	GG	GT	Histological type:squamous cell carcinoma. The primary site:lung
ZNF761	53959151	CC	CA	Histological type:adenocarcinoma. The primary site:esophagus
MED12	70339253	GG	GA	Histological type:leiomyoma. The primary site:smooth muscle

**Table 2:** Analysis of patient's sensitivity to gastrointestinal targeted drugs according to the gene test results

Gene Name	Mutation Code	Standard Gene	Patient's Gene	Mutation Type	Medicine Instruction
BRAF	rs113488022	/	No mutation	/	Sensitive to Cetuximab
KRAS	/	/	No mutation	/	
FCGR3A	rs396991	AA	AC	Heterozygote	
CCND1	rs9344	GG	GA	Heterozygote	
PIK3CA	/	/	No mutation	/	Sensitive to Lapatinib

In conclusion, thorough postoperative pathological examinations of TMT are crucial for tailoring subsequent treatment. Presently, complete resection in conjunction with histologically adapted chemotherapy seems to be suitable and targeted therapy may emerge as an alternative approach.

## Abbreviations

TMT: Teratoma with Malignant Transformation
GCTs: Germ Cell Tumors
CT: Computed Tomography
CK 7: Cytokeratin 7
CK 20: Cytokeratin 20
CEA: Carcino-Embryonic Antigen
CDX2: Caudal-related homeobox gene 2
TTF-1: Thyroid Transcription Factor-1
CK 5/6: Cytokeratin 5/6
WT-1: WilmsTumor gene-1
PFS: Progression-Free Survival
